# Preparation and Characterization of Chitosan/Hydroxypropyl Methylcellulose Temperature-Sensitive Hydrogel Containing Inorganic Salts for Forest Fire Suppression

**DOI:** 10.3390/gels10060390

**Published:** 2024-06-08

**Authors:** Yanni Gao, Yuzhou Zhao, Ting Wang

**Affiliations:** 1 Aulin College, Northeastern Forestry University, Harbin 150040, China; 2021224742@nefu.edu.cn (Y.G.); 1564610285@nefu.edu.cn (Y.Z.); 2 College of Chemistry, Chemical Engineering and Resource Utilization, Northeast Forestry University, 26 Hexing Road, Harbin 150040, China

**Keywords:** forest fire suppression, temperature-sensitive hydrogel, phosphorus salt incorporation, firefighting efficacy

## Abstract

Effective forest fire suppression remains a critical challenge, necessitating innovative solutions. Temperature-sensitive hydrogels represent a promising avenue in this endeavor. Traditional firefighting methods often struggle to address forest fires efficiently while mitigating ecological harm and optimizing resource utilization. In this study, a novel intelligent temperature-sensitive hydrogel was prepared specially for forest fire extinguishment. Utilizing a one-pot synthesis approach, this material demonstrates exceptional fluidity at ambient temperatures, facilitating convenient application and transport. Upon exposure to elevated temperatures, it undergoes a phase transition to form a solid, barrier-like structure essential for containing forest fires. The incorporation of environmentally friendly phosphorus salts into the chitosan/hydroxypropyl methylcellulose gel system enhances the formation of temperature-sensitive hydrogels, thereby enhancing their structural integrity and firefighting efficacy. Morphological and thermal stability analyses elucidate the outstanding performance, with the hydrogel forming a dense carbonized layer that acts as a robust barrier against the spread of forest fires. Additionally, comprehensive evaluations employing rheological tests, cone calorimeter tests, a swelling test, and infrared thermography reveal the multifaceted roles of temperature-sensitive hydrogels in forest fire prevention and suppression strategies.

## 1. Introduction

Forest fires represent a global environmental challenge, characterized by rapid spread and difficulty in containment, posing threats to both ecosystems and human life and property, thus presenting a significant obstacle to economic development [1,2,3]. Traditional fire extinguishing materials, including water, dry powder, and foam, have certain fire extinguishing properties but exhibit limitations in terms of efficiency, ecological impact, and resource utilization [4,5,6]. For instance, while water is widely available and mobile, its high volatility at elevated temperatures limits its effectiveness in reaching the internal high-temperature zones of the flames, leading to substantial water consumption, low economic efficiency [7], and the risk of re-ignition. Similarly, dry powder and foam agents struggle to penetrate the deep-seated fires, inadequately covering extensive fire areas and leaving behind difficult-to-handle resides [8,9].

Contrary to traditional fire extinguishing materials, hydrogels offer promising characteristics such as water retention, adhesion, as well as cooling abilities [10], facilitating flame suppression by providing partial coverage and reducing water consumption, thus enhancing and thus improving firefighting efficiency. Previous studies by Huang et al., Zhou et al., and Wang et al. have demonstrated the efficacy of composite hydrogels in inhibiting combustion and reducing temperatures in various contexts, including coal and mine fires [11,12,13]. However, conventional hydrogels suffer from poor fluidity [14], hindering their penetration capacity and sprayability, resulting in incomplete coverage of ignition points and challenging residue disposal. With advancements in material science, the exploration of novel firefighting materials has gained traction [15,16]. Temperature-sensitive hydrogels, characterized by reversible sol-gel phase transitions in response to temperature changes [17,18,19], offer promising solutions for forest fire management. These smart polymer materials transform into fluid sols below the critical solution temperature (CST) [20], facilitating storage, transportation, and penetration, while transitioning into adhesive gels above this threshold [21], effectively inhibiting oxygen supply and smoke release, thus overcoming limitations of traditional methods.

Cellulose, the most abundant natural polymer compound in the world, serves as the foundation for hydroxypropyl methylcellulose (HPMC), a derivative synthesized via etherification of cellulose [22,23,24]. HPMC incorporates hydrophilic and hydrophobic groups such as hydroxypropyl and methoxy, endowing it with unique hydration–dehydration properties and temperature sensitivity [25]. Its high CST and good water retention make it an ideal candidate for fire extinguishing materials [26,27]. Chitosan (CS), the second most abundant natural polysaccharide derived from deacetylation of chitin, offers biodegradability, non-toxicity, and stability [28,29,30]. It forms excellent gels that are capable of swelling with a variety of liquids, including water [31]. Despite the structural similarities to HPMC, CS features a different molecular weight, which facilitates favorable physical blending for good compatibility and stability. Sodium tripolyphosphate (STPP), an amorphous, water-soluble linear polyphosphate, functions as a moisture-retaining agent and possesses inherent flame-retardant properties, whose addition enhances the formation of a dense carbonized layer [32,33,34]. With synergistic flame-retardant effects to halogenated flame retardants but also being environmentally friendly [35], it is suitable for applications in fire extinguishing materials. Calcium chloride (CaCl_2_) promotes the dehydration process through salting out and interactions, thus accelerating the rate of gel and carbonized layer formation.

**Figure 1:**
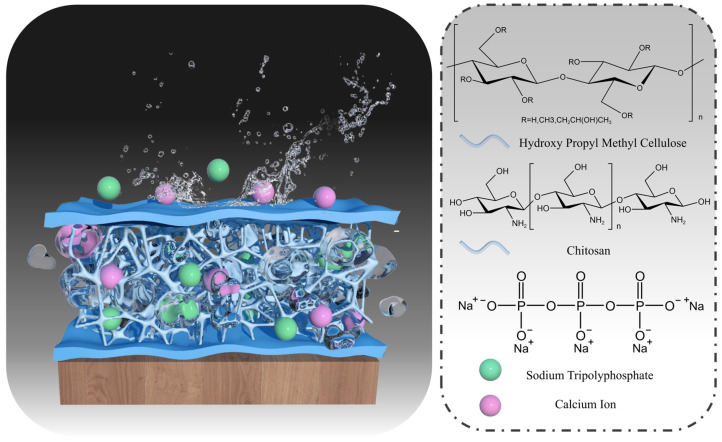
Hydrogel structure adheres to wood substrate and component structure.

This study synthesized a novel temperature-sensitive composite hydrogel based on CS, HPMC, STPP, and calcium chloride (CaCl_2_) by the “one-pot method”, establishing multiple physical cross-linking networks and reversible sol-gel phase transitions (Figure 1). In comparison to chemical covalent cross-linking, physically cross-linking hydrogels are environmentally compatible with natural polymer hydrogels and have no chemical cross-linking agents [36]. Utilizing widely available and environmentally friendly materials, the synthesized fire extinguishing hydrogel exhibits superior properties, including smoke suppression, barrier formation, cooling, temperature sensitivity, water retention, and environmental compatibility. Through comprehensive testing, including rheological, FTIR analysis, swelling test, thermal decomposition, smoke suppression analyses, thermal insulation properties, along with scanning electron microscopy for visualization, this study contributes advanced technical insight and solution strategies for forest fire suppression. 

## 2. Results and Discussion

### 2.1. Gelling Mechanism of Thermosensitive Hydrogels

**Figure 2:**
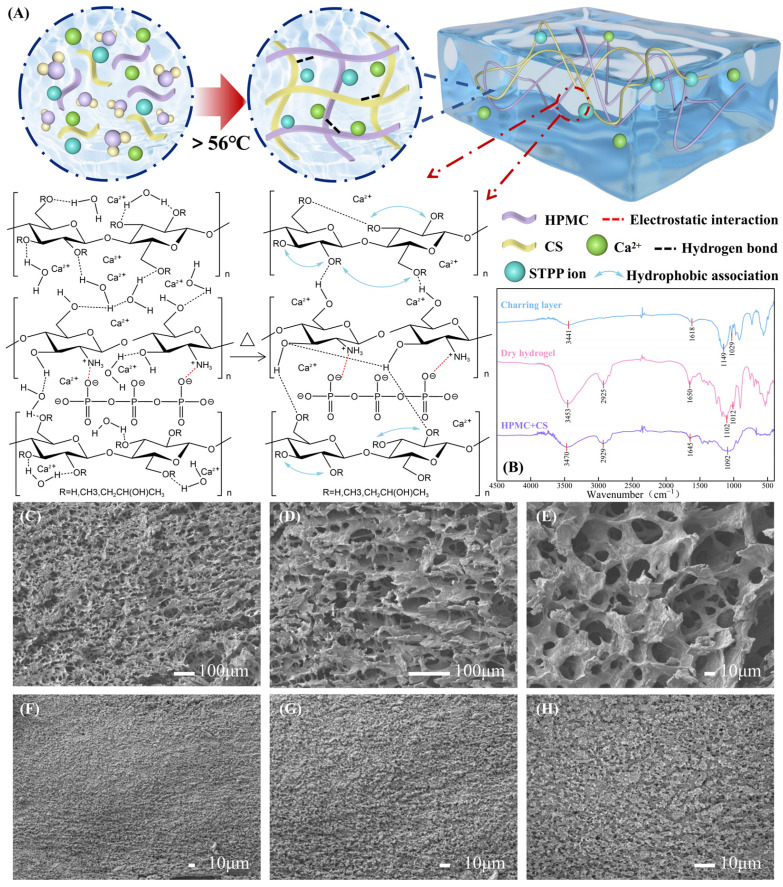
Formation process, network structure, and characterization results of hydrogel. (**A**) Formation of hydrogel physical cross-linking network. (**B**) FTIR spectrum of HPMC + CS, dried hydrogel, and carbonized layer. SEM images of hydrogel network structure, (**C**) ×100, (**D**) ×200, (**E**) ×500. SEM images of the surface of hydrogel carbonization layer b, (**F**) ×300, (**G**) ×500, (**H**) ×1000.

The hydrophilic groups of HPMC, including hydroxyl and hydroxypropyl [25], along with amino groups of chitosan, form hydrogen bonds with water molecules, facilitating their orderly arrangement within the HPMC/CS network. The uniformly dispersed calcium ions in solution and the amino groups of protonated chitosan, interact ionically with polyanionic STPP, forming a structured network (Figure 2A). At lower temperatures, hydrogen bonding between water molecules predominates, leading to a sol state characterized by water coverage [37]. As temperature rises, increased heat absorption disrupts hydrogen bonding, leading to the entanglement of HPMC and CS molecular chains. This, coupled with enhanced salting-out effect, disrupts the ordered water arrangement, gradually exposing hydrophobic methoxylated groups on HPMC. Subsequently, hydrophobic associations dominate, resulting in gel formation.

### 2.2. FTIR Analysis and Swelling Test

Figure 2B shows the FTIR spectra of the HPMC + CS dry blend material, hydrogel, and hydrogel carbonized layer. In the hydrogel system, the peak at 3470 cm^−1^ shifted to a lower wave number region at 3453 cm^−1^, and the intensity of the peak increased compared to the HPMC + CS dry blend material, indicating that more hydrogen bonds are generated in the hydrated hydrogel. In the charcoal layer sample, the peaks at 2800 cm^−1^–3000 cm^−1^ almost disappeared, and the peak at 1149 cm^−1^ shifted to a lower wavelength band, proving that there are fewer C-H bonds in the charcoal layer, but C-C bonds are still present. Both the hydrogel and the charcoal layer samples contained peaks of P-O bonding at 1012 cm^−1^ and 1029 cm^−1^ [38], respectively, whereas the physically dry-mixed powders (HPMC + CS) did not have this characteristic peak. This is consistent with the fact that they do not contain sodium tripolyphosphate and confirms that the carbonized layer contains phosphorus salts. Moreover, the peak near 1650 cm^−1^ represents an in-plane bending vibration of N-H, confirming the presence of chitosan in the system. The absence of new peaks in the hydrogel and charcoal layer spectra proves that the hydrogel is a physical cross-linking system with no chemical reactions occurring. The average swelling ratio of the hydrogel was tested to be 12 times, demonstrating its good water-holding capacity, which is essential for its use as a refractory material.

### 2.3. Structure and Morphology Analysis

The SEM images of the two samples are shown in Figure 2C–H. Figure 2C–E illustrates a hydrogel with a uniformly arranged and dense internal pore network, indicative of a robust three-dimensional interpenetrating structure. This intricate architecture contributes to the hydrogel’s exceptional water retention capabilities. In contrast, Figure 2F–H illustrate the carbon layer formed on the hydrogel after combustion, characterized by a tightly packed surface. This carbon layer not only impedes smoke release but also serves as a barrier, effectively isolating oxygen and inhibiting combustion processes.

### 2.4. Thermal Stability Analysis

**Figure 3:**
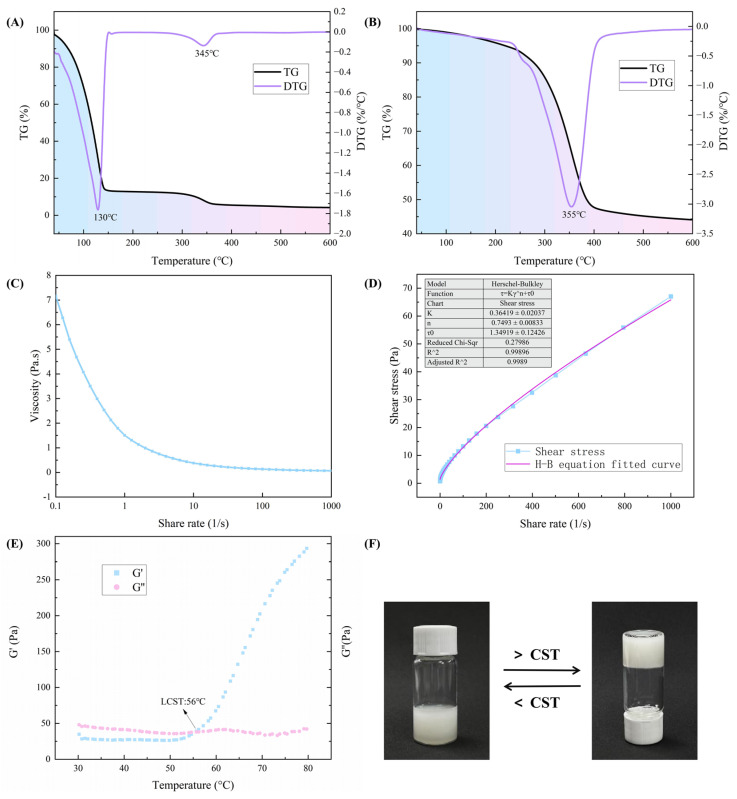
Analysis of thermal stability, rheological properties, modulus, and temperature sensitivity of hydrogels. (**A**) Thermogravimetric curves of aqueous hydrogels. (**B**) Thermogravimetric curves of dried hydrogels. (**C**) Relationship between viscosity and shear rate of hydrogel. (**D**) Relationship between shear stress and shear rate of hydrogel. (**E**) The change of storage modulus and loss modulus of hydrogel with temperature. (**F**) Temperature-sensitive response of hydrogels.

Figure 3 shows the TG and DTG curves of the temperature-sensitive hydrogel, with and without water content, respectively. The pyrolysis process of the temperature-sensitive hydrogel can be divided into two stages by combining the two figures. 

The first weight loss occurred between 40–265 °C and 40–230 °C, with maximum weight loss temperatures of 130 °C and 230 °C, respectively. As can be seen in Figure 3A, the process from 40 °C to 145 °C was dominated by the continuous evaporation of the free water with a weight loss of 84%, which helped to reduce the temperature and dilute the oxygen, and thus mitigating combustion. The dry hydrogel (Figure 3B) had a maximum weight loss temperature of 235 °C and a weight loss of 5% in the initial stage, and this process was mainly the destruction of bound water. 

The second phase of weight loss occurred between 265–453 °C and 235–545 °C, with maximum weight loss temperatures of 345 and 455 °C, respectively, and was dominated by the loss and degradation of some of the polymers and salts. Sodium tripolyphosphate decomposes thermally into sodium phosphate (Na_2_HPO_4_), sodium pyrophosphate (Na_4_P_2_O_7_), and phosphorus dioxide (P_2_O_5_) [39]. At around the maximum weight loss temperature, the gel reaches the peak of the weight loss rate and forms a structurally stable carbonized layer. This carbonized layer acts as a physical barrier to prevent further pyrolysis of the polymer and the release of thermal decomposition products into the gas phase, thus inhibiting combustion and smoke emissions. As the temperature rises, sodium tripolyphosphate and calcium chloride promote dehydration, a process that densifies the carbon layer and further retards combustion of the material [40,41]. Upon reaching 500 °C, the weight remains virtually unchanged. 

Combining the two sets of data, water protects the hydrogel and the dry weight loss of the hydrogel system was about 45% of the initial mass, emphasizing its commendable thermal stability. In summary, the fire extinguishing mechanism can be attributed to the following factors: (Ⅰ) the substantial heat capacity of water facilitates continuous evaporation, resulting in cooling and oxygen dilution. (Ⅱ) STPP acts as a phosphorus flame retardant, promoting carbonization and facilitating the formation of a protective carbonized layer. This layer reduces heat conduction, inhibits oxygen diffusion, and suppresses smoke release, thereby enhancing fire suppression efficacy. 

### 2.5. Investigation of Viscosity

Viscosity is an important index to respond to the fluidity and permeability of hydrogels, which affects the application performance of gels [42,43]. Since hydrogels are subjected to shear in mixing, extrusion, or pumping, it is necessary to explore the relationship between sol viscosity and shear rate using a rotational rheometer [44], and the test results are shown in Figure 3C. From Figure 3C, a consistent decrease in viscosity across all samples with increasing shear rate at room temperature was observed—a phenomenon known as shear thinning.

Significantly, the viscosity reduction is most pronounced at low shear frequencies (*τ* < 1 s^−1^), gradually stabilizing at medium to high shear rates (1 < *τ* < 1000 s^−1^). Shear-thinned fluids offer several advantages, including reduced energy requirements for pumping; enhanced efficiency, particularly in long-distance transport and complex piping systems; and improved spreadability and penetration. Moreover, upon reaching the target site, the viscosity of the hydrogel increases after the external force diminishes, facilitating sample localization and functionality.

### 2.6. Correlation Analysis of Shear Rate and Shear Stress for Hydrogel Performance in Fire Suppression

Investigating the relationship between shear rate and shear stress in hydrogels provides crucial insights into their behavior, particularly concerning their efficacy in fire suppression. Figure 3D illustrates the relationship between shear stress and shear rate of hydrogels at room temperature, with the data fitted using the Herschel–Bulkley equation [45]:

*τ* = *τ*_0_ + *Kγ^n^* (1)

where *τ* is the shear stress (Pa), *τ*_0_ is the static shear stress or the initial shear stress (Pa) of the fluid at zero shear rate, *K* is the fluid flow characteristic, *γ* is the shear rate (s^−1^), and *n* is the rheological index of the fluid, 0 < *n* < 1.

The high correlation coefficient (R^2^ > 0.99) suggests a strong relationship, indicating that the hydrogel behaves as a yield pseudoplastic fluid [46]. This behavior has significant implications, as it implies that the hydrogel does not flow until a critical stress (i.e., the yield stress) is exceeded, highlighting its potential for effective deployment in fire suppression scenarios, where rapid activation is essential.

### 2.7. Correlation of CST Determination with Rheological Properties for Fire Suppression

In forest fire suppression research, the determination of the CST is crucial for understanding the behavior of temperature-sensitive hydrogels. Rheological testing assesses two dynamic moduli: storage modulus (G′), which evaluates sample elasticity, and loss modulus (G″), which measures sample viscosity. The literature indicate that the point where G′ equals G″ signifies the gelling point [47], with the corresponding temperature representing the CST of the hydrogel.

As shown in Figure 3E, the CST of the hydrogel was identified as 56 °C, a critical parameter for effective fire suppression. Below this temperature, G′ < G″, indicating that the sample is in a flowing sol state at this point (Figure 3F), which facilitates penetration and spraying [48]. When the temperature is above the CST, G′ > G″, a phase transition from a sol to an elastic solid occurs [49]. The observed increase in storage modulus with temperature indicates that during heating, the material transforms into the solid gel state and adheres to combustibles to achieve asphyxiation [50], thus highlighting its potential for forest fire mitigation efforts.

### 2.8. Evaluation of Fire Extinguishing and Smoke Suppression Performance of Temperature-Sensitive Hydrogel

**Figure 4:**
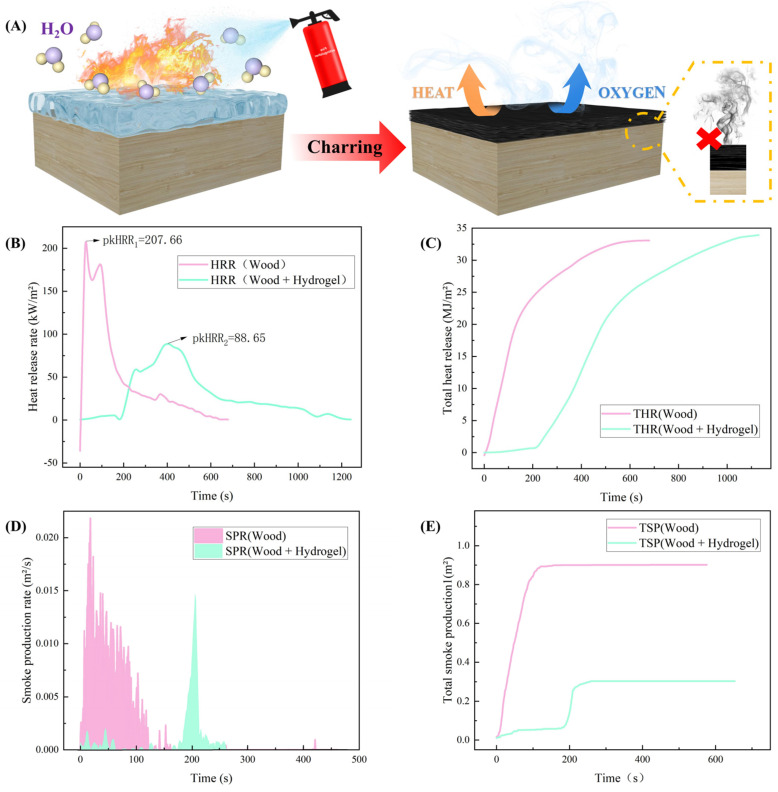
Fire extinguishing performance analysis of hydrogel. (**A**) Hydrogel releases water vapor when heated and the charring layer formed blocks heat and oxygen. Cone calorimeter (**B**) heat release rate. (**C**) Total heat release. (**D**) Smoke production rate. (**E**) Total smoke production.

To comprehensively assess the efficacy of a temperature-sensitive hydrogel as a fire extinguishing material, a cone calorimeter test was conducted. The results, depicted in Figure 4, unveiled significant insights into its performance metrics. Notably, untreated wood panels exhibited a TTI of 9 s, while hydrogel-treated wood displayed a remarkable delay, with TTI extended to 218 s, marking a 24-fold increase. This delay suggests the hydrogel’s formation of a barrier layer on the wood surface, which effectively retards heat transfer and restricts oxygen access, thereby mitigating combustion.

Furthermore, analysis of the HRR demonstrated a noteworthy 57% reduction in peak HRR for hydrogel-treated pine boards compared to dry pine boards, with a significantly prolonged duration to peak HRR occurrence (Figure 4B). This reduction in HRR can be attributed to the hydrogel’s protective film, which impedes heat transfer and suppresses the rapid release of volatile components, thus mitigating flame propagation.

Additionally, the hydrogel exhibited exceptional smoke suppression capabilities, manifesting in a 67% reduction in TSP and significantly decreased SPR for hydrogel-treated pine (Figure 4D,E). This effect is attributed to the hydrogel’s cooling and blocking properties, which hinder the pyrolysis process of wood and reduce the concentration of combustible volatiles in the gas phase. Moreover, as the hydrogel dehydrates, it forms a dense carbonized layer on the wood surface, acting as a physical barrier to inhibit smoke generation and prevent indoor fire asphyxiation.

Based on our research, our temperature-sensitive hydrogel shows great promise as an effective tool for combating forest fires. Our findings highlight its ability to delay ignition and reduce heat release rates, indicating its potential for fire suppression. Additionally, the hydrogel significantly decreased smoke production and release rates, offering further benefits in fire mitigation efforts. Overall, these results underscore the potential of a temperature-sensitive hydrogel in forest fire management strategies.

### 2.9. Assessment of Thermal Regulation and Fire Insulation Performance

**Figure 5:**
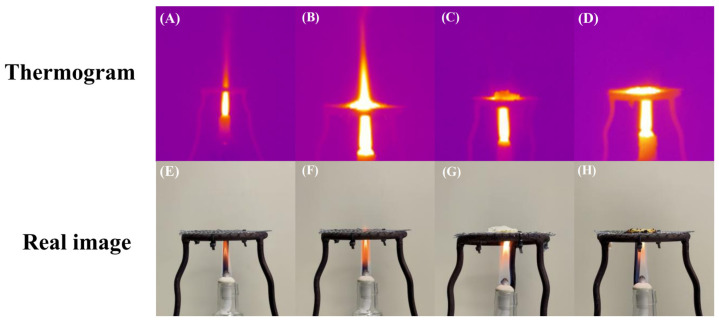
Thermal imaging process diagram of thermal insulation properties of hydrogel. (**A**,**B**) Thermal image of the flame burning gradually increasing. (**C**,**D**) Thermal image of hydrogel placement into carbonization layer. (**E**–**H**) The actual situation corresponding to the thermal images above.

To visually capture the cooling and flame isolation capabilities of the prepared gel, we conducted a series of experiments using a thermal imaging camera. Illustrated in Figure 5A–D are sequential snapshots captured by a thermal imaging camera, documenting the hydrogel’s behavior pre-fire and post-fire extinguishing. Notably, these observations echo our prior findings, underscoring the hydrogel’s gradual water release mechanism that effectively moderates temperature levels. Furthermore, our analysis highlights the gel’s capacity to serve as a thermal barrier, even upon carbonization, reinforcing its potential applications in forest fire mitigation strategies.

## 3. Conclusions

In this study, a novel smart temperature-sensitive hydrogel was prepared by a simple one-pot method for fighting solid fires (e.g., forest fires). The material exhibited excellent fire-fighting properties such as temperature responsiveness, thermal stability, water retention, thermal insulation, and smoke suppression. For example, in cone calorimeter tests, the time to ignition was extended from 9 to 218 s, while the heat release rate and total smoke production were significantly reduced by 57% and 67%, respectively. Thermograms demonstrated the thermal insulation properties of the hydrogel. The extinguishing efficacy of this material can be attributed to several mechanisms. First, its high water content (≥90%) facilitates effective cooling and dilution of combustible gases. Second, above the lower critical solution temperature of 56 °C, the material transforms to a solid state, thereby effectively blocking smoke, heat, and oxygen. Third, the addition of phosphorus-based flame retardants promotes the formation of a dense carbon layer, which further acts as a physical barrier. In conclusion, the multifunctional properties of the temperature-sensitive hydrogel make it a promising candidate for a wide range of applications in the field of fire protection.

## 4. Materials and Methods

### 4.1. Materials

Hydroxypropyl methylcellulose (HPMC, USP2910, 2% viscosity: 3 mPa·s, methoxy: 28–30%; hydroxypropyl: 7.0–12%) was purchased from Shanghai Aladdin Biochemical Science and Technology Co., Ltd. (Shanghai, China). Chitosan (CS, medium viscosity, 200–400 mPa·s) was purchased from Shanghai McLean Biochemical Science and Technology Co., Ltd. (Shanghai, China). Glacial acetic acid (CH_3_CH_2_OH, 99.5%) was purchased from Shanghai Yien Chemical Technology Co., Ltd. (Shanghai, China). Sodium tripolyphosphate (STPP, 99%) was purchased from Shanghai Bidd Pharmaceutical Technology Co., Ltd. (Shanghai, China). Anhydrous calcium chloride purchased from Shanghai Aladdin Biochemical Technology Co., Ltd.

### 4.2. Preparation of Temperature-Sensitive Hydrogels

A 0.2 mol L^−1^ glacial acetic acid solution was prepared and placed in a water bath at 80 °C for 10 min. HPMC and CS powder were accurately weighed and uniformly dry-mixed. The mixture was then slowly added to the glacial acetic acid solution under a constant temperature and continuous stirring until the solute dispersed homogeneously. After cooling to room temperature, a clarified solution was obtained. CaCl_2_ was added and stirred to disperse it evenly in the solution to promote a hydrophobic effect [51]. Then, dropwise STPP was added slowly and stirred, so that it interacts electrostatically with the protonated amino group in chitosan [52,53], enhancing the structure and the flame retardancy of the material, then stirred until a homogeneous white liquid was formed. The ratio of the materials involved was CS:HPMC:STPP:CaCl_2_ = 1:5:3:3, accounting for 9 wt%, and acetic acid accounts for 0.8 wt%.

### 4.3. Temperature-Sensitivity Test

The lower critical solution temperature of the sol-gel was determined using the inverted test tube method [54]. The prepared sol-gel was added to sample bottles and placed in a constant temperature water bath at various temperatures. After 10 min, the bottles were inverted, and fluidity was observed. If the liquid showed no fluidity, it reached its CST; otherwise, the temperature was increased by 1 °C, and the process was repeated until the liquid became non-fluid [55]. The gel formation was confirmed, and changes in sample morphology were observed. The bottle was removed from the water bath, let stand, and watched to see if it changed back to a flowing liquid.

### 4.4. Characterization and Swelling test

The HPMC, CS was dried with the hydrogel by placing it in an oven, and then one portion of the hydrogel was cauterized with an alcohol lamp to a dry carbonized layer. It was characterized by Fourier transform infrared spectroscopy (FTIR, Nicolet IS50, Hefei, China). Scanning electron microscopy (SEM, JSM-7500F, Tokyo, Japan) was performed to observe the internal pores and surface morphology of the hydrogels before (*a*) and after (*b*) combustion. Samples were prepared by freezing one sol-gel in liquid nitrogen and lyophilizing (FD, LGJ-10C, Beijing, China) it, while the other was burned to charcoal. Thermogravimetric analysis (TGA, NETZSCH STA 449F3, Georgenthal, Germany) was performed on both water-containing and oven-dried hydrogel samples in a nitrogen atmosphere with a temperature range of 25–600 °C and a temperature increase rate of 10 °C min^−1^. The average swelling ratio of the hydrogel was tested to be 12 times, which responds to the good water holding capacity of the hydrogel. Hydrogels must retain a large amount of water to be used as a refractory material.

### 4.5. Rheological Characteristics

The rheological characterization of the samples was performed by rotational rheometer (RheolabQC, AR2000ex, Newcastle, USA). The viscosity as a function of shear rate and the relationship between shear stress and shear rate were measured at room temperature (25 °C). Additionally, the storage modulus (G′) and loss modulus (G″) as a function of temperature during gel formation were measured over a temperature range of 30–90 °C with a temperature increase rate of 5 °C min^−1^ and a frequency of 1 Hz.

### 4.6. Combustion Performance Test

Pine boards (100 mm × 100 mm × 4 mm) were used and dried in a vacuum oven for 24 h. The mass difference between the two boards after drying was in the range of ±0.1 g. One of the pine boards was immersed in a beaker of temperature-sensitive hydrocolloid for 10 min, and the dried pine boards were wrapped in aluminum foil according to the ISO5660-1 standard using a cone calorimeter (CONE, 6810, Suzhou, China). One of the pine boards was evenly covered with a 1.5 mm thick layer of sol-gel sample. The sample cassettes were then placed 25 mm away from the cone and the time to ignition (TTI) as well as the heat release rate (HRR), the total heat release (THR), the smoke production rate (SPR), and the total smoke production (TSP) of the samples were measured at a radiation intensity of 50 kW m^−2^.

### 4.7. Insulation and Cooling Performance Test

An alcohol lamp was placed below a large porous mesh support table, and an infrared thermal imager was used to record temperature changes before and after placing the temperature-sensitive hydrogel in its gel state, assessing its thermal insulation and cooling effects.

### 4.8. Statistical Analysis

Statistical methods were used to compare the differences between groups and evaluate their statistical significance. The results were analyzed as mean standard deviation. The statistical analysis was carried out with Office and Origin 2021 software. The experimental results of mechanistic reasoning were explained and analyzed by drawing schematic diagrams using C4D, Office, and Photoshop. The significance level was set to * *p* < 0.05, indicating statistical significance; ** *p* < 0.01 denoted a high statistical significance; *** *p* < 0.001 signified the highest level of statistical significance [56].

## Data Availability

The original contributions presented in the study are included in the article; further inquiries can be directed to the corresponding authors.

## References

[1] Warneke C., Schwarz J.P., Dibb J., Kalashnikova O., Frost G., Al-Saad J., Brown S.S., Brewer W.A., Soja A., Seidel F.C. (2023). Fire influence on regional to global environments and air quality (firex-aq). J. Geophys. Res.-Atmos..

[2] Jones M.W., Abatzoglou J.T., Veraverbeke S., Andela N., Lasslop G., Forkel M., Smith A.J.P., Burton C., Betts R.A., van der Werf G.R. (2022). Global and regional trends and drivers of fire under climate change. Rev. Geophys..

[3] Zacharakis I., Tsihrintzis V.A. (2023). Environmental forest fire danger rating systems and indices around the globe: A review. Land.

[4] Dubocq F., Bjurlid F., Ydstål D., Titaley I.A., Reiner E., Wang T., Almirall X.O., Kärrman A. (2020). Organic contaminants formed during fire extinguishing using different firefighting methods assessed by nontarget analysis. Environ. Pollut..

[5] Bokka S., Ameta P., Achary S.N., Chowdhury A. (2024). A simple and economical fire test setup for examining the fire retardancy/extinguishing ability of water additive fire-retardant materials on class a fires. Fire Mater..

[6] Son M.-H., Kim Y., Jo Y.-H., Kwon M. (2021). Assessment of chemical asphyxia caused by toxic gases generated from rigid polyurethane foam (rpuf) fires. Forensic Sci. Int..

[7] Loschilov A.A. (2023). Simplified mathematical model of inhibition of exothermic process in modeling the extinguishing of a forest fire. Fluid Dyn..

[8] Viriyawattana N., Sinworn S. (2023). Performance improvement of the dry chemical-based fire extinguishers using nanocalcium silicate synthesised from biowaste. J. Fire Sci..

[9] Arvidson M., Mindykowski P. (2023). Fire testing of alternative fixed fire-extinguishing systems for ro-ro spaces onboard ships. Ships Offshore Struct..

[10] Zhang Y., Wu B.M. (2023). Current advances in stimuli-responsive hydrogels as smart drug delivery carriers. Gels.

[11] Huang Z.A., Yan L.K., Zhang Y.H., Gao Y.K., Liu X.H., Liu Y.Q., Li Z.Y. (2019). Research on a new composite hydrogel inhibitor of tea polyphenols modified with polypropylene and mixed with halloysite nanotubes. Fuel.

[12] Zhou C.S., Tang Y.B. (2018). A novel sodium carboxymethyl ellulose/aluminium citrate gel for extinguishing spontaneous fire in coal mines. Fire Mater..

[13] Wang C.Y., Shi H., Wang X., Song L., Hu Y. (2023). Carrageenan-vermiculite-dimethyl methyl phosphate ternary hybrid hydrogels for firefighting. Fire Mater..

[14] Irfan M., Shah L.A., Khan A., Farooq M., Ullah M., Ismail M. (2023). Formulation of zwitter-ionic terpolymeric hydrogels and their comprehensive rheological investigation. J. Dispers. Sci. Technol..

[15] Yllmaz-Atay H., Wilk-Jakubowski J.L. (2022). A review of environmentally friendly approaches in fire extinguishing: From chemical sciences to innovations in electrical engineering. Polymers.

[16] Pathak T.K., Sharma V., Jassal P.S., Singh R.P., Johar R. (2022). Bio-modified pyrotechnic composite materials for firefighting application. Fire Mater..

[17] Tanaka M., Nakahata M., Linke P., Kaufmann S. (2020). Stimuli-responsive hydrogels as a model of the dynamic cellular microenvironment. Polym. J..

[18] Dethe M.R., Prabakaran A., Ahmed H., Agrawal M., Roy U., Alexander A. (2022). Pcl-peg copolymer based injectable thermosensitive hydrogels. J. Control. Release.

[19] El-Husseiny H.M., Mady E.A., Hamabe L., Abugomaa A., Shimada K., Yoshida T., Tanaka T., Yokoi A., Elbadawy M., Tanaka R. (2022). Smart/stimuli-responsive hydrogels: Cutting-edge platforms for tissue engineering and other biomedical applications. Mater. Today Bio.

[20] Chelu M., Musuc A.M. (2023). Polymer gels: Classification and recent developments in biomedical applications. Gels.

[21] Huang C., Dai Z., Jiang Z., Chen Y., Zhong M. (2024). Wood stack fire tests to evaluate the influence of extinguishing medium and driving pressure on fire extinguishing efficacy of forest trees. Therm. Sci. Eng. Prog..

[22] Koochaki A., Shahgholi M., Sajadi S.M., Babadi E., Inc M. (2023). Investigation of the mechanical stability of polyethylene glycol hydrogel reinforced with cellulose nanofibrils for wound healing: Molecular dynamics simulation. Eng. Anal. Bound. Elem..

[23] Saddik M.S., Elsayed M.M.A., El-Mokhtar M.A., Sedky H., Abdel-Aleem J.A., Abu-Dief A.M., Al-Hakkani M.F., Hussein H.L., Al-Shelkamy S.A., Meligy F.Y. (2022). Tailoring of novel azithromycin-loaded zinc oxide nanoparticles for wound healing. Pharmaceutics.

[24] Pargaonkar S.S., Ghorpade V.S., Mali K.K., Dias R.J., Havaldar V.D., Kadam V.J., Pargaonkar M.S.S. (2023). Hydrogel films of citric acid cross-linked hydroxypropyl methylcellulose/methylcellulose for hydrophilic drug delivery. Indian J. Pharm. Educ. Res..

[25] Chiaregato C.G., Bernardinelli O.D., Shavandi A., Sabadini E., Petri D.F.S. (2023). The effect of the molecular structure of hydroxypropyl methylcellulose on the states of water, wettability, and swelling properties of cryogels prepared with and without cao2. Carbohydr. Polym..

[26] Carvalho J.D.D., Rabelo R.S., Hubinger M.D. (2022). Thermo-rheological properties of chitosan hydrogels with hydroxypropyl methylcellulose and methylcellulose. Int. J. Biol. Macromol..

[27] Yang Y., Liang Z., Zhang R., Zhou S., Yang H., Chen Y., Zhang J., Yin H., Yu D. (2024). Research advances in superabsorbent polymers. Polymers.

[28] Do N.H.N., Truong Q.T., Le P.K., Ha A.C. (2022). Recent developments in chitosan hydrogels carrying natural bioactive compounds. Carbohydr. Polym..

[29] Furlani F., Rossi A., Grimaudo M.A., Bassi G., Giusto E., Molinari F., Lista F., Montesi M., Panseri S. (2022). Controlled liposome delivery from chitosan-based thermosensitive hydrogel for regenerative medicine. Int. J. Mol. Sci..

[30] Thirupathi K., Raorane C.J., Ramkumar V., Ulagesan S., Santhamoorthy M., Raj V., Krishnakumar G.S., Phan T.T.V., Kim S.C. (2023). Update on chitosan-based hydrogels: Preparation, characterization, and its antimicrobial and antibiofilm applications. Gels.

[31] Yudaev P., Semenova A., Chistyakov E. (2024). Gel based on modified chitosan for oil spill cleanup. J. Appl. Polym. Sci..

[32] Abdel-Hakim A., El-Basheer T.M., Abdelkhalik A. (2020). Mechanical, acoustical and flammability properties of sbr and sbr-pu foam layered structure. Polym. Test..

[33] Witono A.I., Zheng X., Saito Y., Noro S.I. (2024). Shaping of metal-organic framework using chitosan and triphosphate cross-linker. Chem. Lett..

[34] Vazquez C.I.H., Draczynski Z., Borkowski D., Kazmierczak D. (2024). Enhancing chitosan fibers: A dual approach with tripolyphosphate and ursolic acid. Polymers.

[35] Venier M., Salamova A., Hites R.A. (2015). Halogenated flame retardants in the great lakes environment. Acc. Chem. Res..

[36] Yang J., Chen Y., Zhao L., Zhang J., Luo H. (2023). Constructions and properties of physically cross-linked hydrogels based on natural polymers. Polym. Rev..

[37] Niemczyk-Soczynska B., Sajkiewicz P., Gradys A. (2022). Toward a better understanding of the gelation mechanism of methylcellulose via systematic dsc studies. Polymers.

[38] Alaoui Y., Laourayed M., Er-rafai A., Hammi M., El Moudane M., Boudalia M., Sekkat Z., Warad I., Guenbour A., Bellaouchou A. (2022). Effect of alumina insertion on structural properties, thermal stability, and chemical durability of potassium calcium based-phosphate glasses. Inorg. Chem. Commun..

[39] Banach M., Makara A. (2011). Thermal decomposition of sodium phosphates. J. Chem. Eng. Data.

[40] Zhang H., Tian J., Yan L., Zhou S., Liang M., Zou H. (2023). Improving the ablation properties of liquid silicone rubber composites by incorporating hexaphenoxycyclotriphosphonitrile. Nanomaterials.

[41] Zhou S., Song L., Wang Z., Hu Y., Xing W. (2008). Flame retardation and char formation mechanism of intumescent flame retarded polypropylene composites containing melamine phosphate and pentaerythritol phosphate. Polym. Degrad. Stab..

[42] Roy K., Lee D.H., Ryplida B., In I., Bhang S.H., Park S.Y. (2023). A self-reporting mineralized conductive hydrogel sensor with cancer-selective viscosity, adhesiveness, and stretchability. Adv. Funct. Mater..

[43] Lee D.H., Madsen E.A., Linnes J.C., Wereley S.T. (2023). Temporally and spatially resolved micro-rheometry of a transient viscous polymer formation. Meas. Sci. Technol..

[44] Goncu Y., Ay N. (2023). Boron nitride’s morphological role in the design of injectable hyaluronic acid based hybrid artificial synovial fluid. Acs Biomater. Sci. Eng..

[45] Lee M.Y.J., Shin K., Nam J. (2023). Operating limit of vacuum-assisted slot die coating of herschel-bulkley fluids. Chem. Eng. Sci..

[46] Marum D.M., Afonso M.D., Ochoa B.B. (2020). Rheological behavior of a bentonite mud. Appl. Rheol..

[47] Samiei M., Abdollahinia E.D., Amiryaghoubi N., Fathi M., Barar J., Omidi Y. (2023). Injectable thermosensitive chitosan/gelatin hydrogel for dental pulp stem cells proliferation and differentiation. Bioimpacts.

[48] Safronov A.P., Rusinova E.V., Terziyan T.V., Zemova Y.S., Kurilova N.M., Beketov I.V., Zubarev A.Y. (2023). Gelation in alginate-based magnetic suspensions favored by poor interaction among sodium alginate and embedded particles. Appl. Sci..

[49] Kim E.J., Choi J.S., Kim J.S., Choi Y.C., Cho Y.W. (2016). Injectable and thermosensitive soluble extracellular matrix and methylcellulose hydrogels for stem cell delivery in skin wounds. Biomacromolecules.

[50] González Enriquez N.D., Patiño-Herrera R., Catarino-Centeno R., Palestino G., Almendárez Camarillo A., Pérez E. (2023). Hydrophobization of hair to improve the interfacial adhesion between hair and high-density polyethylene. Polym. Eng. Sci..

[51] Wu W.M., Hou J.R., Li G., Chen L.F. (2022). Effect of temperature and inorganic salts concentration on syneresis rate of am/dac hydrogel. Colloids Surf. A-Physicochem. Eng. Asp..

[52] Çakir M.A., Icyer N.C., Tornuk F. (2020). Optimization of production parameters for fabrication of thymol-loaded chitosan nanoparticles. Int. J. Biol. Macromol..

[53] Hamidi F., Aghdam M.A., Johar F., Mehdinejad M.H. (2022). Ionic gelation synthesis, characterization and adsorption studies of cross-linked chitosan-tripolyphosphate (cs-tpp) nanoparticles for removal of as (v) ions from aqueous solution: Kinetic and isotherm studies. Toxin Rev..

[54] Haider M.S., Ahmad T., Yang M.S., Hu C., Hahn L., Stahlhut P., Groll J. (2021). Tuning the thermogelation and rheology of poly(2-oxazoline)/poly(2-oxazine)s based thermosensitive hydrogels for 3d bioprinting. Gels.

[55] Boonrat O., Tantishaiyakul V., Hirun N. (2022). Micellization and gelation characteristics of different blends of pluronic f127/methylcellulose and their use as mucoadhesive in situ gel for periodontitis. Polym. Bull..

[56] Azam F., Ahmad F., Ahmad S., Zafar M.S., Ulker Z. (2023). Synthesis and characterization of natural fibers reinforced alginate hydrogel fibers loaded with diclofenac sodium for wound dressings. Int. J. Biol. Macromol..

